# Do Diagnosis Related Groups change admission practice to a large Swiss ICU?

**DOI:** 10.1186/cc12422

**Published:** 2013-03-19

**Authors:** K Homburg, CA Rüst, P Fodor, S Blumenthal

**Affiliations:** 1 Triemli City Hospital, Zurich, Switzerland

## Introduction

The new Swiss financing system by means of Diagnosis Related Groups (DRG), effective 1 January 2012, forms the basis of a performance-based system to reimburse general hospital services. There are only few data on the influence of DRG on ICU patients [1,2]. We expect that the introduction of the DRG in Switzerland will change the number of admissions from external hospitals to a large ICU with a centre function and will influence the severity of disease of the admitted patients.

## Methods

The ICU of the Triemli City Hospital in Zurich has an interdisciplinary organisation with surgical and internal medical patients, with a maximum occupancy of 18 beds and a centre function for the surrounding hospitals of the region. In this prospective ongoing observational study, we collect and analyse the anonymised data of all patients admitted to our ICU from an external hospital during 12 months prior to (1 January to 31 December 2011) and after (1 January to 31 December 2012) the introduction of the DRG in Switzerland. Exclusion criteria are admissions by the emergency department, self-assignments into the hospital and internal relocations. The primary endpoint is the number of admissions from an external hospital to our ICU. Secondary endpoints are the severity of the disease of the admitted patients, detected by the scoring systems SAPS II and APACHE II as well as the length of stay in external hospitals before admission. The statistical analysis is descriptive.

## Results

We present the preliminary data for 10 months (in each case January to October) before and after the introduction of the DRG. We observed an increase of 9.2% (391 vs. 427 patients) of admissions to our ICU after the introduction of the DRG. The severity of disease determined by the SAPS II score is unchanged (mean 26.7 vs. 26.0 points, *P *= 0.466). The severity of disease determined by the APACHE II score is significantly lower (15.4 vs. 14 points, *P *= 0.017). We also noted that after the introduction of the DRG the patients were earlier transferred from an external hospital to our ICU (mean time until transfer 29.9 vs. 18.7 hours), but this value was not significant (*P *= 0.55).

## Conclusion

Up to now the introduction of the DRG in Switzerland has had a complex influence on the number and the kind of patients admitted from an external hospital to an ICU with centre function. Data assessment and analysis will continue.
